# The role of diet and periodontitis in preserved ratio impaired spirometry: a mediation analysis

**DOI:** 10.3389/fnut.2026.1834131

**Published:** 2026-05-29

**Authors:** Hao Sun, Taolin Zhang, Ziqian Song, Yuantao Qi

**Affiliations:** 1 Qingdao Municipal Hospital, University of Health and Rehabilitation Sciences, Qingdao, China; 2 Department of Epidemiology and Health Statistics, School of Public Health, Cheeloo College of Medicine, Shandong University, Jinan, China; 3 Shandong Cancer Hospital and Institute, Shandong First Medical University and Shandong Academy of Medical Sciences, Jinan, China

**Keywords:** DII, mediation analysis, OBS, oxidative stress, periodontitis, PRISM, systemic inflammation

## Abstract

**Background:**

Preserved ratio impaired spirometry (PRISm) is an early form of lung dysfunction linked to systemic inflammation and oxidative stress. While diet and periodontitis independently affect pulmonary health, their interrelationship and potential mediating pathways remain unclear.

**Objective:**

To explore the cross-sectional associations between dietary inflammatory indices, periodontitis, and PRISm, and to examine whether periodontitis statistically accounts for part of the diet-PRISm association. An external validation analysis was conducted to assess reproducibility rather than to confirm causality or definitive generalizability.

**Methods:**

This cross-sectional analysis used data from the National Health and Nutrition Examination Survey (NHANES, 2009–2012), including 3,540 participants aged ≥30 years with complete periodontal, spirometric, dietary, laboratory, and covariate information. The Dietary Inflammatory Index (DII) and Oxidative Balance Score (OBS) were used to assess dietary inflammatory potential and oxidative balance, while periodontitis was defined according to CDC/AAP criteria. PRISm was identified based on spirometry results. Mediation analysis was performed as an exploratory statistical decomposition of associations, not as evidence of temporal or causal mediation. An external hospital-based cohort of 1,126 participants from the Health Examination Center of the Second Affiliated Hospital of Shandong First Medical University, Shandong Province, China, was used to evaluate the reproducibility of the main findings.

**Results:**

In NHANES, higher DII values were associated with greater odds of PRISm (OR = 1.17, 95% CI: 1.06–1.29), while higher OBS values were associated with lower odds of PRISm (OR = 0.96, 95% CI: 0.93–0.99). Periodontitis was independently associated with PRISm (OR = 1.61, 95% CI: 1.04–2.50). Mediation analysis suggested that periodontitis accounted for only a small statistical proportion of the DII-PRISm association (6.46%) and the OBS-PRISm association (9.58%). In the external validation cohort, OBS remained inversely associated with PRISm (OR = 0.92, 95% CI: 0.84-0.99), whereas the DII-PRISm association was directionally positive but not statistically significant (OR = 1.18, 95% CI: 0.93–1.49).

**Conclusions:**

Pro-inflammatory diets, lower oxidative balance, and periodontitis were associated with PRISm odds in NHANES, and periodontitis accounted for a small statistical proportion of the observed diet-PRISm associations. The external cohort supported the OBS pattern, whereas DII-related findings were not statistically significant and should not be considered externally confirmed.

## 1 Introduction

PRISm, represents a common but understudied pulmonary pattern. Population-based estimates place its prevalence around 6–17% globally (1, 2). Individuals with PRISm often exhibit increased respiratory symptoms, reduced exercise capacity, higher systemic inflammation, and elevated morbidity and mortality relative to persons with normal lung function (3–5). Notably, PRISm has been linked to metabolic and cardiovascular comorbidities: higher body mass index, diabetes, cardiovascular disease, and tobacco smoke exposure are consistently associated with this phenotype (6–10). Collectively, these findings implicate chronic systemic inflammation and oxidative stress as key mechanistic pathways underlying PRISm, highlighting the potential role of modifiable lifestyle factors such as diet and oral health.

Dietary patterns modulate systemic inflammation and oxidative stress, influencing chronic disease risk. The DII is a validated literature-derived score that quantifies the inflammatory potential of a person's diet (11, 12). Higher DII values reflect pro-inflammatory diets, whereas lower values indicate anti-inflammatory diets (13). Prior work has linked higher DII scores with elevated systemic inflammatory markers (e.g., IL-6) and poorer lung function. For example, in asthma patients, a pro-inflammatory diet was associated with higher odds of asthma, lower FEV1, and increased IL-6 levels (14). Conversely, an anti-inflammatory diet is thought to attenuate chronic inflammation and may protect pulmonary health (15). The OBS complements the DII by summarizing cumulative antioxidant and pro-oxidant exposures from diet and lifestyle. Higher OBS values indicate a predominance of antioxidant (e.g., vitamins C, E, β-carotene, and fiber) over pro-oxidant (e.g., saturated fat, iron, and alcohol) exposures (16). A recent NHANES analysis demonstrated that higher OBS was associated with better lung function (higher FEV1, FVC) and lower odds of chronic bronchitis, wheezing, or restrictive spirometric patterns (15, 17). These findings highlight how nutrient-rich, low-inflammatory diets may support respiratory health, while pro-oxidant diets might impair it.

Periodontitis is a common chronic inflammatory disease of the gums, characterized by destruction of tooth-supporting tissues. In the US, roughly 40% of adults ≥30 years old have some degree of periodontitis, and prevalence exceeds 60% in those over age 65. Periodontitis is more than an oral disease; it triggers local and systemic inflammation and oxidative stress (18). Bacterial biofilms in periodontal pockets stimulate neutrophils and other immune cells to release reactive oxygen species and pro-inflammatory cytokines, leading to tissue damage locally and raised systemic markers (e.g., CRP and IL-6) (19, 20). Epidemiological studies link periodontitis to systemic conditions such as cardiovascular disease, diabetes, and even neurodegeneration (21). Importantly, recent meta-analyses indicate a positive association between periodontitis and chronic respiratory diseases: periodontitis increases the odds of chronic obstructive pulmonary disease (COPD) and obstructive sleep apnea, and is related to worse outcomes in pneumonia and COVID-19 (22). Periodontitis-associated systemic inflammation is thus considered a modifiable risk factor for poor lung health.

Emerging evidence suggests that diet, periodontitis, and lung function may be interrelated via inflammatory pathways. Pro-inflammatory diets have been shown to raise periodontitis risk, while antioxidant-rich diet was inversely associated with gum disease (23, 24). A large Korean cohort study reported that individuals consuming the most pro-inflammatory diets had significantly higher incidence of periodontitis than those with anti-inflammatory diets (25). Similarly, a recent NHANES analysis found that higher OBS was linked to markedly lower odds of periodontitis. In turn, oral infections like periodontitis could exacerbate systemic inflammation and promote lung pathology.

To evaluate the reproducibility of our findings, we also conducted an external validation analysis using an independent hospital-based dataset from Shandong Province, China. This validation analysis was intended to examine whether the main directions and statistical evidence of association observed in NHANES could be observed in a different clinical population, rather than to provide definitive proof of generalizability. Because both datasets were observational and cross-sectional in analytic structure, the study was designed to generate hypotheses and to describe association patterns, not to establish causality or temporal mediation.

## 2 Materials and methods

### 2.1 Study design and sample

The NHANES component of this study was an observational cross-sectional analysis. Exposure, mediator, and outcome information were measured within the same survey framework; therefore, the temporal order among diet, periodontitis, and PRISm could not be established.

**Figure 1:**
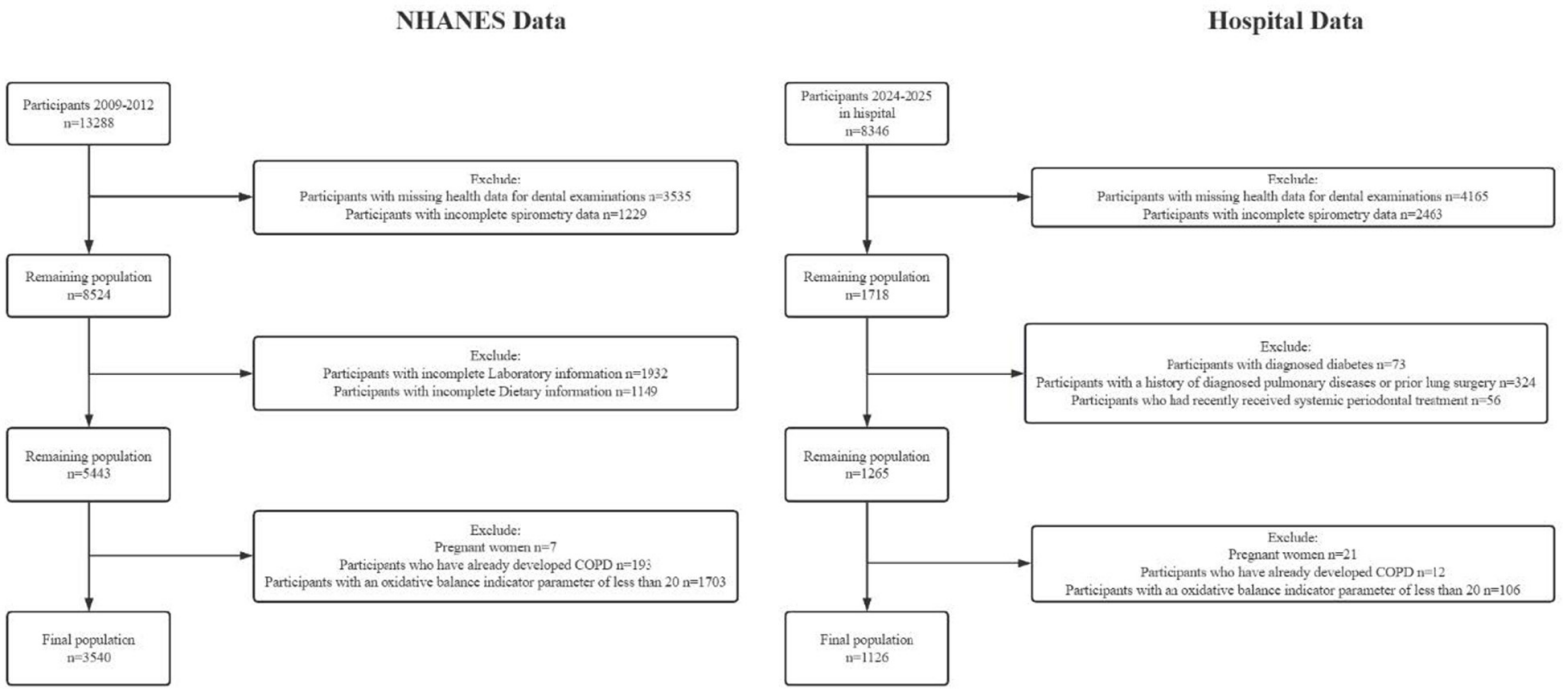
Flowchart for screening the study population.

This study was based on data from NHANES 2009–2012. Initially, a total of 13,288 participants were considered. After excluding individuals with missing dental examination data (*n* = 3,535) and those with incomplete spirometry data (*n* = 1,229), 8,524 participants remained. Further exclusions were applied for incomplete laboratory information (*n* = 1,932) and incomplete dietary information (n = 1,149), resulting in 5,443 participants. Finally, pregnant women (*n* = 7), individuals with pre-existing chronic obstructive pulmonary disease (COPD) (*n* = 193), and participants with incomplete information for the 20 OBS components (*n* = 1,703) were excluded. The OBS was constructed from 20 predefined dietary, lifestyle, and biomarker components; therefore, missing information for any component would prevent calculation of a valid total OBS and could introduce measurement error. Accordingly, this exclusion was applied as a complete-component requirement for OBS construction rather than as an exclusion based on a low OBS score. The detailed OBS components and scoring criteria are provided in Supplementary Table S1. The final analytical sample consisted of 3,540 participants. The participant selection process is illustrated in Figure 1.

To externally evaluate the reproducibility of the findings derived from NHANES, an independent hospital-based validation cohort was established. This cohort included adults who underwent routine health examinations at the Health Examination Center of the Second Affiliated Hospital of Shandong First Medical University, Shandong Province, China, between June 2024 and December 2025.

After institutional screening for available dental examination, spirometry, dietary, and covariate information, and after excluding participants with diagnosed pulmonary disease or prior lung surgery, pregnancy, pre-existing COPD, recent systemic periodontal treatment, and incomplete information for the 20 OBS components (*n* = 106), the final external validation cohort comprised 1,126 participants. The same complete-component requirement was applied because OBS could not be calculated reliably when any of its 20 constituent indicators was unavailable. The detailed OBS components and scoring criteria are provided in Supplementary Table S1.

This external dataset was used to assess the reproducibility of the main associations observed in NHANES, especially the OBS and periodontitis findings. Because this was a hospital-based cohort with different population structure and measurement context, the validation results were interpreted cautiously.

### 2.2 Sources of core variables

DII and OBS were selected as key dietary exposure indicators because they comprehensively capture the inflammatory and oxidative balance of diet, reflecting two major biological pathways of systemic inflammation and oxidative stress that are implicated in both periodontal disease and pulmonary function impairment.

Data for calculating DII and OBS were extracted from multiple NHANES datasets. The DII was calculated according to the literature-derived method of Shivappa et al. (26). Briefly, each available dietary component was standardized using the global reference mean and standard deviation, converted to a centered percentile score, multiplied by the corresponding inflammatory effect score, and summed to obtain the overall DII. Because NHANES does not contain all 45 original DII parameters, only components available from the 24 h dietary recall and total nutrient intake files were used, including energy, carbohydrate, protein, total fat, saturated fat, monounsaturated fatty acids, polyunsaturated fatty acids, n-3 fatty acids, n-6 fatty acids, cholesterol, fiber, alcohol, caffeine, β-carotene, thiamin, riboflavin, niacin, vitamin B6, folate, vitamin B12, vitamins A, C, D, and E, iron, magnesium, zinc, and selenium. Unavailable DII components were not imputed. The OBS was calculated by scoring available antioxidant-favorable and pro-oxidant exposures from dietary, laboratory, and questionnaire data. Antioxidant components were scored from low to high exposure, whereas pro-oxidant components were reverse scored; component scores were then summed, with higher OBS values indicating a more antioxidant-favorable balance. Participants missing variables required to derive the final DII or OBS were excluded rather than imputed.

Periodontal status was assessed by trained dentists following the NHANES periodontal examination protocol. Periodontitis was classified according to the Centers for Disease Control and Prevention/American Academy of Periodontology (CDC/AAP) case definition. Participants were categorized into four groups: no periodontitis, mild, moderate, and severe periodontitis. Mild, moderate, and severe periodontitis were collectively defined as periodontitis (“yes”), while “no periodontitis” was used as the reference category (27). In the external validation cohort, periodontal status was recorded by dental clinicians using the same four-level severity structure (no, mild, moderate, and severe periodontitis), and the binary variable was harmonized in the same manner. Because the validation cohort was hospital-based, differences in the examination setting and diagnostic workflow are acknowledged as a limitation.

Spirometry measurements were conducted using standardized NHANES procedures following American Thoracic Society guidelines. PRISm was defined as a forced expiratory volume in one second to forced vital capacity ratio (FEV_1_/FVC) ≥0.70 combined with FEV1 < 80% of the predicted value. The primary analysis used this fixed-ratio definition to maintain comparability with prior epidemiological PRISm studies, and participants not meeting PRISm criteria were treated as the reference group.

### 2.3 Covariate

Covariates were selected based on previous studies and biological plausibility. The following variables were included in the adjusted models: age, sex, race/ethnicity (non-Hispanic White, non-Hispanic Black, Mexican American, other Hispanic, other races), marital status (married/living with partner vs. unmarried), body mass index (BMI), poverty-to-income ratio (PIR), and total energy intake (kcal/day).

Comorbidities included hypertension, diabetes mellitus, and cardiovascular disease (CVD). Hypertension was defined as systolic blood pressure ≥140 mmHg, diastolic blood pressure ≥90 mmHg, use of antihypertensive medication, or self-reported diagnosis. Diabetes was identified by fasting glucose ≥126 mg/dL, HbA1c ≥6.5%, use of glucose-lowering medication, or self-reported diagnosis. CVD included self-reported coronary heart disease, heart failure, angina, or myocardial infarction.

### 2.4 Statistical analyses

All analyses accounted for the NHANES complex, multistage probability sampling design using appropriate survey weights, strata, and primary sampling units to generate nationally representative estimates.

Baseline characteristics were compared between participants with and without PRISm using the χ^2^ test for categorical variables, Student's *t*-test for normally distributed continuous variables, and Wilcoxon rank-sum test for skewed distributions.

Multivariable logistic regression models were employed to estimate odds ratios (ORs) and 95% confidence intervals (CIs) for the associations between DII, OBS, and PRISm, DII, OBS and periodontitis, as well as between periodontitis and PRISm. Models were sequentially adjusted for potential confounders. Model 1 adjusted for age sex, and race; Model 2 additionally adjusted for marital status, PIR, BMI, and total energy intake; Model 3 further adjusted for diabetes, hypertension, and CVD. Survey-weighted linear regression was used to examine associations between dietary inflammation indices and the mediators using the same model-building strategy.

Non-linearity between continuous dietary inflammation indices and the log-odds of PRISm was tested using restricted cubic splines (RCS) with 3 knots in fully adjusted logistic regression models. Additional stratified analyses were conducted based on periodontitis status. To address potential misclassification related to the fixed FEV1/FVC threshold, we performed a sensitivity analysis in NHANES using a lower-limit-of-normal (LLN) definition for FEV1/FVC based on NHANES III/Hankinson reference equations (28), while retaining FEV1 < 80% predicted as the impairment criterion. This LLN sensitivity analysis was not performed in the validation cohort because a validated race/ethnicity-specific LLN equation and complete predicted-value parameters were not available in the analytical validation dataset.

Mediation analysis was conducted to quantify whether periodontitis statistically accounted for a portion of the total association between diet and PRISm. The analysis used a product-of-coefficients approach within a counterfactual framework, with bootstrapping (1000 iterations) for the indirect effect. All mediation models were adjusted for all covariates in Model 3. Because diet, periodontitis, and PRISm were measured cross-sectionally, the indirect effects were interpreted as exploratory decompositions under model assumptions, not as evidence that periodontitis temporally or causally transmits dietary effects on PRISm.

Analyses were performed using R software (version 4.4.3) and a two-sided *p*-value < 0.05 indicated statistical significance.

### 2.5 External validation analysis

The external validation analysis used the same exposure–outcome framework as the primary NHANES analysis. Logistic regression models assessed the associations of DII, OBS, and periodontitis with PRISm, as well as the associations of DII and OBS with periodontitis.

Model 1 adjusted for age and sex. Model 2 additionally adjusted for marital status, BMI, socioeconomic status (SES), smoking status, and total energy intake when available. Model 3 further adjusted for hypertension, diabetes, and CVD. Race/ethnicity and poverty-income ratio were not included because they were unavailable in the external validation dataset.

Mediation analysis in the external cohort evaluated periodontitis as a potential statistical mediator between dietary indices and PRISm, using the same analytic framework as the primary NHANES analysis where applicable. These validation mediation results were interpreted only as supportive or non-supportive statistical decompositions, because the external cohort also lacked longitudinal temporality.

Unlike NHANES, the external validation cohort did not have complex survey weights; therefore, all validation analyses were conducted using standard regression methods. Validation findings were interpreted as replicated only when both the direction and statistical evidence were compatible with the NHANES results.

## 3 Results

### 3.1 Characteristics of the included population

A total of 3,540 participants were included, of whom 4.96% had PRISm (Table 1). Participants with PRISm were more likely to be Non-Hispanic Black (*P* < 0.01) and to have a higher prevalence of hypertension (*P* = 0.01), diabetes (*P* < 0.01), and cardiovascular disease (*P* = 0.02) compared with those without PRISm. They also exhibited a higher mean BMI (*P* < 0.01) and poorer oxidative balance (*P* < 0.01), along with a more pro-inflammatory diet (*P* < 0.01). In an additional obesity-focused description requested during peer review, obesity (BMI >30 kg/m^2^) was more common among NHANES participants with PRISm than among those without PRISm (61.3% vs. 32.7%, *P* < 0.01). Periodontitis was significantly more prevalent among individuals with PRISm (*P* < 0.01). No significant differences were observed in age, sex distribution, smoking behavior, or total energy intake between groups.

**Table 1:** Baseline characteristics of the NHANES data.

Variable	Preserved ratio impaired spirometry			
	Total	No	Yes	*P* value
Total	3, 540 (100.00)	3, 333 (95.04)	207 (4.96)	
Age years	49.14 (0.34)	49.11 (0.36)	49.56 (1.06)	0.70
**Sex %**				0.61
Female	1, 672 (47.83)	1, 564 (47.72)	108 (50.04)	
Male	1, 868 (52.17)	1, 769 (52.28)	99 (49.96)	
**Race %**				< 0.01
Non-Hispanic White	1, 697 (74.59)	1, 619 (75.03)	78 (66.21)	
Non-Hispanic Black	688 (8.94)	614 (8.43)	74 (18.73)	
Mexican American	478 (6.56)	470 (6.78)	8 (2.43)	
Other Hispanic	331 (4.39)	324 (4.54)	7 (1.50)	
Other race	346 (5.52)	306 (5.22)	40 (11.13)	
**Marital status**				0.04
Married/Living with partner	2, 383 (71.83)	2, 250 (72.14)	133 (65.79)	
Widowed/Divorced/Separated	741 (17.86)	687 (17.44)	54 (25.91)	
Never married	416 (10.31)	396 (10.41)	20 (8.30)	
Family PIR	3.37 (0.06)	3.38 (0.06)	3.11 (0.16)	0.08
BMI kg/m^2^	28.63 (0.16)	28.48 (0.15)	31.62 (0.59)	< 0.01
**Smoking behavior %**				0.74
Never	1, 976 (56.74)	1, 865 (56.90)	111 (53.64)	
Former	918 (27.16)	865 (27.11)	53 (28.03)	
Now	646 (16.10)	603 (15.99)	43 (18.33)	
Energy intake kcal	2, 235.30 (26.42)	2, 238.20 (26.45)	2, 179.73 (73.36)	0.41
**Hypertension %**				0.01
No	2, 163 (64.91)	2, 061 (65.43)	102 (54.81)	
Yes	1, 377 (35.09)	1, 272 (34.57)	105 (45.19)	
**Diabetes %**				< 0.01
No	3, 000 (88.74)	2, 853 (89.47)	147 (74.74)	
Yes	540 (11.26)	480 (10.53)	60 (25.26)	
**Cardiovascular disease %**				0.02
No	3, 355 (95.46)	3, 170 (95.73)	185 (90.18)	
Yes	185 (4.54)	163 (4.27)	22 (9.82)	
OBS	22.86 (0.15)	22.96 (0.14)	21.07 (0.58)	< 0.01
DII	1.00 (0.05)	0.97 (0.05)	1.52 (0.15)	< 0.01
**Periodontitis %**				< 0.01
No	1, 783 (59.33)	1, 701 (59.99)	82 (46.74)	
Mild	245 (6.24)	223 (5.85)	22 (13.71)	
Moderate	1, 124 (26.63)	1, 045 (26.29)	79 (33.05)	
Severe	388 (7.80)	364 (7.87)	24 (6.50)	

We analyzed a total of 1,126 participants in the external validation cohort. The validation cohort differed from NHANES in population source and covariate structure, and the results were therefore interpreted as a reproducibility assessment rather than definitive generalization. Individuals with PRISm showed higher prevalence of hypertension (*P* < 0.01) and diabetes (*P* = 0.01), and periodontitis was more prevalent in the PRISm group (*P* < 0.01), consistent with the direction observed in NHANES. In contrast to NHANES, obesity prevalence was not higher among participants with PRISm in the validation cohort (9.6% vs. 10.8%, *P* = 0.86). The demographic and clinical characteristics of the external cohort are shown in Table 2.

**Table 2:** Baseline characteristics of the validation data.

**Variable**	Preserved ratio impaired spirometry			
	Total	No	Yes	*P* value
Total	1, 126 (100.00)	1, 037 (92.10)	89 (7.90)	
Age years	48.92 (0.36)	48.66 (0.38)	52.02 (1.21)	0.01
**Sex %**				0.79
Female	553 (49.11)	511 (49.28)	42 (47.19)	
Male	573 (50.89)	526 (50.72)	47 (52.81)	
**Marital status**				0.17
Divorced/Widowed	118 (10.48)	113 (10.90)	5 (5.62)	
Married/Partner	795 (70.60)	725 (69.91)	70 (78.65)	
Single	213 (18.92)	199 (19.19)	14 (15.73)	
SES index	5.53 (0.07)	5.51 (0.07)	5.78 (0.23)	0.28
BMI kg/m^2^	25.30 (0.11)	25.26 (0.12)	25.82 (0.39)	0.17
Energy intake kcal	2, 162.32 (17.80)	2, 161.57 (18.71)	2, 170.90 (57.06)	0.88
OBS	22.38 (0.10)	22.41 (0.10)	22.07 (0.35)	0.35
DII	1.00 (0.03)	0.99 (0.04)	1.12 (0.12)	0.33
**Smoking behavior %**				0.62
Current	313 (27.80)	285 (27.48)	28 (31.46)	
Former	151 (13.41)	138 (13.31)	13 (14.61)	
Never	662 (58.79)	614 (59.21)	48 (53.93)	
**Hypertension %**				< 0.01
No	809 (71.85)	757 (73.00)	52 (58.43)	
Yes	317 (28.15)	280 (27.00)	37 (41.57)	
**Diabetes %**				0.01
No	941 (83.57)	876 (84.47)	65 (73.03)	
Yes	185 (16.43)	161 (15.53)	24 (26.97)	
**Cardiovascular disease %**				0.83
No	1, 062 (94.32)	979 (94.41)	83 (93.26)	
Yes	64 (5.68)	58 (5.59)	6 (6.74)	
**Periodontitis severity**				< 0.01
No	702 (62.34)	662 (63.84)	40 (44.94)	
Mild	86 (7.64)	67 (6.46)	19 (21.35)	
Moderate	245 (21.76)	226 (21.79)	19 (21.35)	
Severe	93 (8.26)	82 (7.91)	11 (12.36)	

### 3.2 Relationship between DII, OBS, and PRISm

As shown in Table 3, higher DII values were significantly associated with higher odds of PRISm across all models. In the fully adjusted model (Model 3), each unit increase in DII was associated with 17% higher odds of PRISm (OR = 1.17, 95% CI: 1.06–1.29, *P* < 0.01). Compared with the lowest quartile (Q1), participants in the highest quartile (Q4) of DII had approximately twofold higher odds of PRISm (OR = 2.04, 95% CI: 1.13–3.71, *P* = 0.02). In contrast, higher OBS was associated with lower odds of PRISm. Each one-point increase in OBS was related to 4% lower odds of PRISm (OR = 0.96, 95% CI: 0.93–0.99, *P* = 0.01), and those in the highest quartile of OBS exhibited nearly 45% lower odds of PRISm compared with Q1 (OR = 0.55, 95% CI: 0.31–0.97, *P* = 0.04).

**Table 3:** Association between DII, OBS, and PRISm.

Inflammation index	Model 1		Model 2		Model 3	
	OR (95% CI)	*P* value	OR (95% CI)	*P* value	OR (95% CI)	*P* value
**NHANES data**						
**DII**						
Continuous	1.18 (1.07, 1.30)	< 0.01	1.17 (1.06, 1.29)	< 0.01	1.17 (1.06, 1.29)	< 0.01
Q1	Reference					
Q2	1.13 (0.61, 2.07)	0.69	1.11 (0.64, 1.92)	0.71	1.08 (0.59, 1.98)	0.80
Q3	2.04 (1.18, 3.51)	0.01	1.94 (1.18, 3.20)	0.01	1.91 (1.11, 3.27)	0.02
Q4	2.17 (1.21, 3.88)	0.01	2.10 (1.19, 3.68)	0.01	2.04 (1.13, 3.71)	0.02
OBS						
Continuous	0.96 (0.93, 0.98)	< 0.01	0.96 (0.93, 0.98)	< 0.01	0.96 (0.93, 0.99)	0.01
Q1	Reference					
Q2	1.15 (0.71, 1.86)	0.57	1.13 (0.68, 1.86)	0.63	1.15 (0.71, 1.86)	0.55
Q3	0.63 (0.33, 1.18)	0.14	0.64 (0.32, 1.26)	0.19	0.66 (0.34, 1.30)	0.22
Q4	0.50 (0.28, 0.89)	0.02	0.50 (0.28, 0.89)	0.02	0.55 (0.31, 0.97)	0.04
**Validation data**						
DII						
Continuous	1.11 (0.91, 1.34)	0.30	1.17 (0.93, 1.47)	0.19	1.18 (0.93, 1.49)	0.17
Q1	Reference					
Q2	1.15 (0.61, 2.16)	0.67	1.09 (0.51, 2.35)	0.82	1.19 (0.55, 2.57)	0.66
Q3	1.05 (0.56, 1.99)	0.88	0.93 (0.42, 2.06)	0.86	0.97 (0.44, 2.17)	0.95
Q4	1.38 (0.75, 2.54)	0.30	1.61 (0.79, 3.28)	0.19	1.66 (0.80, 3.41)	0.17
OBS						
Continuous	0.97 (0.91, 1.04)	0.36	0.92 (0.85, 1.00)	0.05	0.92 (0.84, 0.99)	0.04
Q1	Reference					
Q2	0.90 (0.51, 1.58)	0.71	0.90 (0.48, 1.71)	0.76	0.91 (0.48, 1.74)	0.78
Q3	0.49 (0.27, 0.90)	0.02	0.36 (0.17, 0.77)	< 0.01	0.36 (0.17, 0.77)	< 0.01
Q4	0.83 (0.44, 1.56)	0.56	0.52 (0.23, 1.21)	0.13	0.47 (0.20, 1.11)	0.09

In the external validation cohort, DII showed a positive but statistically non-significant association with PRISm (OR = 1.18, 95% CI: 0.93–1.49, *P* = 0.17). The inverse association between OBS and PRISm was replicated, with each unit increase in OBS associated with 8% lower odds of PRISm (OR = 0.92, 95% CI: 0.84–0.99, *P* = 0.04). These findings indicate that the OBS-PRISm association was externally supported, whereas the DII-PRISm association was not statistically reproduced and should be interpreted as less robust.

### 3.3 Relationship between DII, OBS, and periodontitis

As shown in Table 4, a higher DII was positively associated with the prevalence of periodontitis in NHANES. In the fully adjusted model (Model 3), each one-unit increase in DII was associated with 9% higher odds of periodontitis (OR = 1.09, 95% CI: 1.02–1.17, *P* = 0.02). Compared with participants in the lowest DII quartile (Q1), those in the highest quartile (Q4) had higher odds of periodontitis (OR = 1.75, 95% CI: 1.17–2.62, *P* = 0.01), suggesting a graded association pattern. Conversely, OBS was inversely associated with periodontitis. Each one-unit increase in OBS was linked to 5% lower odds of periodontitis (OR = 0.95, 95% CI: 0.94–0.97, *P* < 0.01), and individuals in the highest quartile exhibited 56% lower odds of periodontitis compared with Q1 (OR = 0.44, 95% CI: 0.33–0.59, *P* < 0.01).

**Table 4:** Association between DII, OBS, and periodontitis.

Inflammation index	Model 1		Model 2		Model 3	
	OR (95% CI)	*P* value	OR (95% CI)	*P* value	OR (95% CI)	*P* value
**NHANES data**						
**DII**						
Continuous	1.07 (1.01, 1.14)	0.03	1.09 (1.02, 1.17)	0.01	1.09 (1.02, 1.17)	0.02
Q1	Reference					
Q2	1.26 (0.97, 1.63)	0.08	1.31 (1.03, 1.66)	0.03	1.29 (1.01, 1.65)	0.04
Q3	1.21 (0.93, 1.58)	0.15	1.33 (0.99, 1.80)	0.06	1.32 (0.97, 1.81)	0.08
Q4	1.58 (1.10, 2.25)	0.01	1.77 (1.19, 2.63)	0.01	1.75 (1.17, 2.62)	0.01
OBS						
Continuous	0.96 (0.95, 0.98)	< 0.01	0.95 (0.94, 0.97)	< 0.01	0.95 (0.94, 0.97)	< 0.01
Q1	Reference					
Q2	0.71 (0.56, 0.90)	0.01	0.66 (0.52, 0.84)	< 0.01	0.66 (0.52, 0.84)	< 0.01
Q3	0.60 (0.42, 0.85)	0.01	0.52 (0.38, 0.72)	< 0.01	0.52 (0.38, 0.72)	< 0.01
Q4	0.53 (0.41, 0.70)	< 0.01	0.43 (0.33, 0.58)	< 0.01	0.44 (0.33, 0.59)	< 0.01
**Validation data**						
DII						
Continuous	0.98 (0.88, 1.09)	0.67	0.93 (0.82, 1.06)	0.30	0.94 (0.82, 1.07)	0.33
Q1	Reference					
Q2	1.13 (0.80, 1.59)	0.49	0.96 (0.64, 1.44)	0.83	0.98 (0.65, 1.48)	0.93
Q3	1.04 (0.74, 1.47)	0.81	0.93 (0.62, 1.40)	0.72	0.94 (0.62, 1.42)	0.78
Q4	0.89 (0.63, 1.25)	0.49	0.73 (0.48, 1.10)	0.13	0.73 (0.48, 1.10)	0.14
OBS						
Continuous	0.92 (0.89, 0.96)	< 0.01	0.93 (0.89, 0.97)	< 0.01	0.92 (0.88, 0.97)	< 0.01
Q1	Reference					
Q2	0.76 (0.55, 1.07)	0.11	0.79 (0.53, 1.18)	0.25	0.79 (0.53, 1.18)	0.26
Q3	0.68 (0.50, 0.93)	0.02	0.72 (0.49, 1.04)	0.08	0.72 (0.49, 1.04)	0.08
Q4	0.46 (0.31, 0.68)	< 0.01	0.52 (0.33, 0.82)	< 0.01	0.50 (0.31, 0.79)	< 0.01

In the external validation cohort, the DII association with periodontitis was not statistically significant and did not replicate the NHANES finding (OR = 0.94, 95% CI: 0.82–1.07, *P* = 0.33). However, OBS remained significantly inversely associated with periodontitis (OR = 0.92, 95% CI: 0.88–0.97, *P* < 0.01), with the highest OBS quartile showing lower odds of periodontitis compared with the lowest quartile. Thus, the validation cohort supported the OBS-periodontitis association but not the DII-periodontitis association.

### 3.4 Relationship between periodontitis and PRISm

As presented in Table 5, the presence of periodontitis was positively associated with the odds of PRISm. In the crude model (Model 1), individuals with periodontitis showed significantly higher odds of PRISm compared with those without periodontitis (OR = 1.81, 95% CI: 1.18–2.78, *P* = 0.01). After adjustment for potential confounders including demographic, lifestyle, and metabolic factors (Model 3), the association remained statistically significant but slightly attenuated (OR = 1.61, 95% CI: 1.04–2.50, *P* = 0.03). The categorical analysis further demonstrated a similar direction, where higher severity of periodontal disease corresponded to elevated odds of PRISm, although the trend was no longer statistically significant after full adjustment (*P* = 0.14).

**Table 5:** Association between periodontitis and PRISm.

Independent variable	Model 1		Model 2		Model 3	
	OR (95% CI)	*P* value	OR (95% CI)	*P* value	OR (95% CI)	*P* value
**NHANES data**						
Categorical variable	1.18 (1.02, 1.36)	0.03	1.12 (0.97, 1.29)	0.11	1.11 (0.96, 1.27)	0.14
Periodontitis						
No	Reference					
Yes	1.81 (1.18, 2.78)	0.01	1.64 (1.06, 2.53)	0.03	1.61 (1.04, 2.50)	0.03
Validation data						
Categorical variable	1.23 (1.02, 1.50)	0.03	1.35 (1.07, 1.69)	0.01	1.32 (1.05, 1.66)	0.02
Periodontitis						
No	Reference					
Yes	2.08 (1.34, 3.23)	< 0.01	2.96 (1.72, 5.09)	< 0.01	2.84 (1.65, 4.89)	< 0.01

In the external validation cohort, the presence of periodontitis was associated with higher odds of PRISm, with individuals with periodontitis showing more than double the odds of PRISm compared with those without periodontitis (OR = 2.84, 95% CI: 1.65–4.89, *P* < 0.01). The severity score of periodontitis was also associated with higher odds of PRISm (OR = 1.32, 95% CI: 1.05–1.66, *P* = 0.02). Because the validation cohort was hospital-based, the larger estimate may reflect population composition, measurement differences, and unmeasured confounding rather than a truly larger biological effect.

### 3.5 Non-linear relationship between DII, OBS, and PRISm

**Figure 2:**
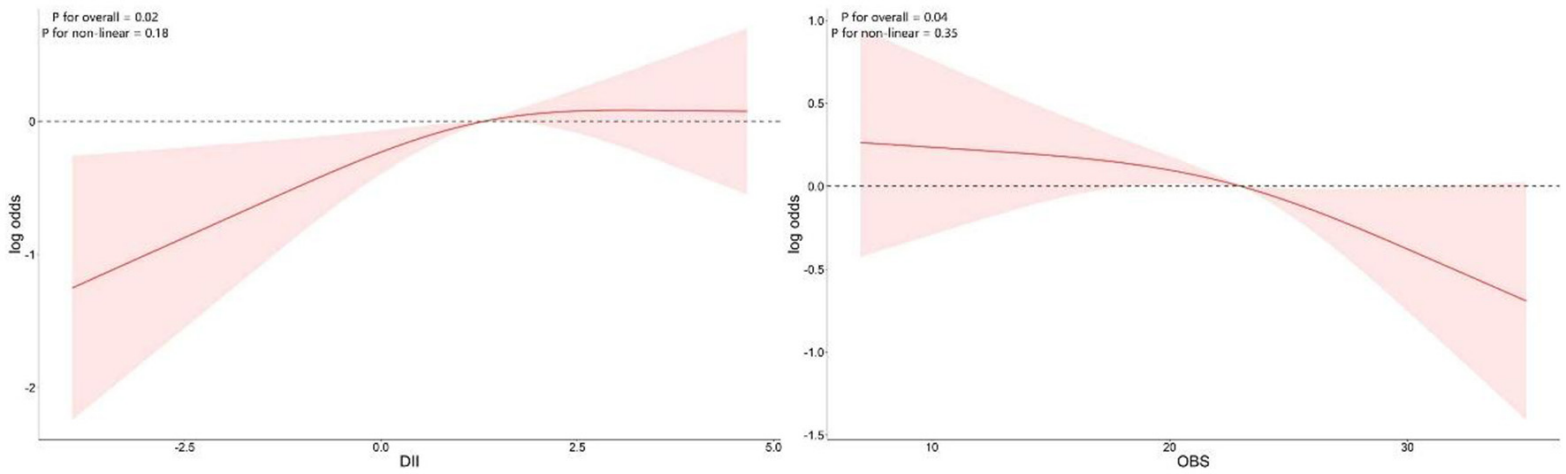
RCS showing the relationship between DII, OBS, and odds of PRISm (NHANES data).

**Figure 3:**
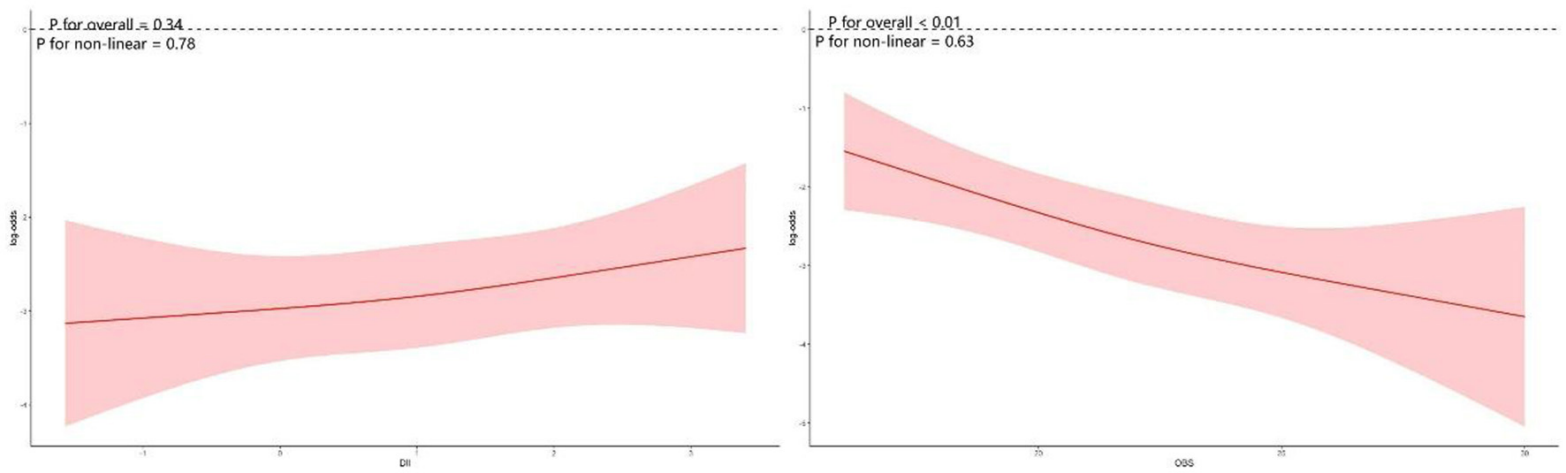
RCS showing the relationship between DII, OBS, and odds of PRISm (Validation data).

Figure 2 presents the results of the non-linear analysis between the inflammation indices and PRISm. After adjustment for all covariates, a significant positive linear association was observed between DII and the odds of PRISm (*P* for overall = 0.02; *P* for non-linear = 0.18). Conversely, a significant inverse association was found for OBS in relation to the odds of PRISm (*P* for overall = 0.04; *P* for non-linear = 0.35) (Figure 3).

### 3.6 Mediation analysis

**Figure 4:**
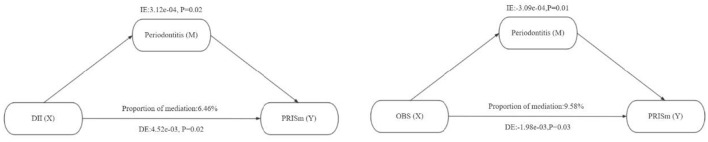
Statistical mediation analysis of periodontitis in the associations of DII and OBS with PRISm (NHANES data).

**Figure 5:**
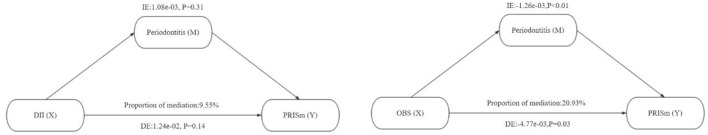
Statistical mediation analysis of periodontitis in the associations of DII and OBS with PRISm (Validation data).

In both the NHANES and external validation datasets, periodontitis was assessed as a potential statistical mediator in the association between DII, OBS, and PRISm (Figures 4, 5).

For DII, the NHANES mediation analysis showed a statistically significant but small indirect association through periodontitis. The indirect effect (IE) was 3.12e-04 (*P* = 0.02), accounting for 6.46% of the total association, with a direct effect (DE) of 4.52e-03 (*P* = 0.02). In the external validation cohort, the indirect effect for the DII-PRISm association was not statistically significant (IE = 1.08e-03, *P* = 0.31), consistent with the non-significant DII associations in that cohort. Therefore, the DII-related mediation result should be regarded as exploratory and not externally confirmed.

For OBS, NHANES analysis showed a statistically significant but partial indirect association through periodontitis, with an indirect effect of −3.09e-04 (*P* = 0.01), explaining 9.58% of the total association, and a direct effect of −1.98e-03 (*P* = 0.03). In the external validation cohort, the OBS-related indirect association was also statistically significant (IE = −1.26e-03, *P* < 0.01), accounting for 20.93% of the total association. The larger mediation proportion in the validation cohort should be interpreted cautiously because of differences in population source, periodontal assessment, covariate availability, and the distribution of OBS and periodontitis; it should not be interpreted as evidence of a stronger causal pathway.

### 3.7 Sensitivity analysis

In a sensitivity analysis using an LLN-based PRISm definition in NHANES, 292 participants were classified as having PRISm. The OBS association remained inverse and statistically significant (OR = 0.96, 95% CI: 0.94–0.99, *P* = 0.01). The DII association remained positive but became borderline (OR = 1.10, 95% CI: 1.00–1.21, *P* = 0.05), and the periodontitis association was attenuated and no longer statistically significant (OR = 1.26, 95% CI: 0.82–1.93, *P* = 0.30). Thus, the LLN sensitivity analysis supported the direction of the dietary findings, particularly OBS, but suggested that the periodontitis-PRISm association may be sensitive to the spirometric definition (Table 6).

**Table 6:** Sensitivity analysis using LLN-defined PRISm in NHANES data.

Exposure	OR (95% CI)^*^	*P* value
DII	1.10 (1.00, 1.21)	0.05
OBS	0.96 (0.94, 0.99)	0.01
Periodontitis		
No	Reference	
Yes	1.26 (0.82, 1.93)	0.30

^*^Models were adjusted for age, sex, race/ethnicity, marital status, PIR, BMI, total energy intake, hypertension, diabetes, and CVD. OR, odds ratio; CI, confidence interval; LLN, lower limit of normal.

## 4 Discussion

In this nationally representative cross-sectional study, we found that DII, OBS, and periodontitis were associated with PRISm in NHANES, and that periodontitis statistically accounted for a small proportion of the diet-PRISm associations. These results are consistent with prior evidence linking dietary inflammatory potential and oxidative balance with lung-function-related outcomes, as well as evidence connecting periodontal inflammation with respiratory diseases (15, 21, 22, 29). However, the external validation results were mixed: the OBS-PRISm and periodontitis-PRISm associations were supported, whereas the DII-PRISm association and DII-related mediation were not statistically significant.

The DII-related findings warrant particular caution. Although DII was associated with PRISm and periodontitis in NHANES, the corresponding associations were not statistically significant in the external cohort, and the DII-periodontitis estimate was not directionally consistent. These discrepancies may reflect differences in population source, sample size, dietary assessment context, covariate availability, and residual confounding. Accordingly, DII should be viewed as a less robust signal in this study, whereas the OBS-related associations were more consistently reproduced.

Pro-inflammatory diets often contain abundant saturated fats, refined carbohydrates, and low fiber, which have been linked in prior mechanistic studies to inflammatory signaling such as NF-κB activation and cytokine production (30–32). In the present study, however, we did not directly measure these signaling pathways. Therefore, the mechanistic interpretation should be viewed as biologically plausible but indirect. Poor diet may also be associated with periodontal inflammation, and prior epidemiologic studies have reported associations between dietary inflammatory potential, oxidative balance, and periodontal outcomes (21, 23, 25, 33). Our NHANES results showed that higher DII was associated with periodontitis, while higher OBS was inversely associated with periodontitis. The external cohort replicated the OBS–periodontitis association but not the DII–periodontitis association, suggesting that dietary inflammation findings may be more context-sensitive than oxidative balance findings.

In PRISm, one hypothesis is that low-grade systemic inflammation contributes to small-airway or parenchymal changes despite a preserved FEV1/FVC ratio; this hypothesis is conceptually consistent with literature on inflammatory and oxidative pathways in lung injury, oxidative stress dynamics, and pulmonary fibrosis (34–36). Our data are compatible with this hypothesis but cannot directly test it because harmonized inflammatory biomarkers such as CRP, IL-6, or oxidative stress indicators were not available across the full analytic NHANES cycle set used in this study and were not available in the uploaded validation dataset. Accordingly, we have tempered statements regarding NF-κB signaling, ROS, and systemic cytokines, and we avoid presenting these pathways as directly demonstrated by our data.

The oral–lung axis remains a plausible explanatory framework (22, 37, 38). Periodontal pathogens or their products may be aspirated into the airway, and periodontal inflammation may contribute to systemic immune activation, potentially involving neutrophil-driven reactive oxygen species and inflammatory pathways (38, 39). Nevertheless, our study did not measure airway microbiota, aspiration events, surfactant biology, pulmonary imaging, or circulating inflammatory mediators. Therefore, statements about periodontal inflammation contributing to lung tissue stiffness, surfactant alteration, or subclinical fibrosis have been reframed as hypotheses requiring longitudinal and mechanistic confirmation.

The mediation findings also require conservative interpretation. A statistically significant indirect effect in cross-sectional data does not demonstrate that diet preceded periodontitis or that periodontitis preceded PRISm. Such analyses further rely on strong assumptions, including no unmeasured exposure-outcome, exposure-mediator, or mediator-outcome confounding and correct model specification. Therefore, the mediation estimates should be interpreted as quantitative descriptions of association patterns rather than proof of an etiologic pathway.

From a clinical perspective, the observed effect sizes should be interpreted cautiously. The per-unit associations for DII and OBS were modest, and the mediation proportions were small in NHANES. Although the validation cohort showed a larger periodontitis-PRISm estimate, the hospital-based design, different measurement context, and potential residual confounding limit direct clinical translation. Dietary counseling and periodontal screening remain low-risk strategies with broad health benefits, but our findings do not establish that modifying diet or treating periodontitis will prevent PRISm. Future longitudinal and interventional studies are required before clinical recommendations specific to PRISm can be made.

This study has several notable strengths. First, it is based on a large, nationally representative NHANES sample with standardized spirometry, dentist-performed periodontal examinations, and validated dietary recalls. Second, two complementary dietary indices were used to capture inflammatory and oxidative dietary patterns, with OBS construction following previously used scoring approaches based on antioxidant and pro-oxidant components (40). Third, survey-weighted regression, mediation analysis, RCS analyses, obesity-focused supplementary analyses, and an LLN-based PRISm sensitivity analysis were incorporated during revision. The external validation cohort provided an additional assessment of reproducibility, although it should be interpreted as partial rather than definitive validation.

Nevertheless, several limitations should be acknowledged. First, the cross-sectional design precludes causal inference and raises the possibility of reverse causality. Participants with early pulmonary dysfunction may have changed their diet, physical activity, dental-care behavior, or medical-care utilization after respiratory symptoms or comorbidities developed. Second, although extensive adjustments were made, residual confounding from unmeasured factors such as environmental pollutants, occupational exposures, oral-hygiene behavior, medication use, or healthcare access cannot be ruled out. Third, the mediation analysis depends on assumptions that cannot be verified in the present data, including adequate control of exposure-mediator, mediator-outcome, and exposure-outcome confounding and correct model specification. Fourth, dietary intake was derived from 24 h recall data, which may not fully reflect habitual consumption and is subject to recall bias. Fifth, because OBS required complete information on all 20 constituent components, the complete-component requirement led to exclusion of participants with incomplete OBS data; this may have reduced sample size and may limit generalizability if excluded participants differed systematically from those included. Sixth, inflammatory and oxidative biomarkers were not consistently available across the full analytic dataset, so mechanistic explanations remain indirect. Seventh, the external cohort was hospital-based and differed from NHANES in population structure and measurement context. Finally, PRISm was primarily defined using a fixed FEV1/FVC threshold; although we added an LLN-based sensitivity analysis in NHANES, some associations—particularly periodontitis—were attenuated under the LLN definition.

## 5 Conclusion

Our study suggests that diet, oxidative balance, periodontitis, and PRISm are associated in cross-sectional data, and that periodontitis may account for a small statistical proportion of the observed diet-PRISm associations. The OBS-related findings were more consistently reproduced than the DII-related findings. These results should be interpreted as hypothesis-generating rather than causal. Future longitudinal and interventional studies incorporating periodontal treatment, dietary modification, and direct inflammatory or oxidative biomarkers are needed to clarify temporal and causal pathways.

## Data Availability

The raw data supporting the conclusions of this article will be made available by the authors, without undue reservation.
