# Supplementary material for: The role of diet and periodontitis in preserved ratio impaired spirometry: a mediation analysis
Source: Front Nutr. 2026 May 29;13:1834131. doi: 10.3389/fnut.2026.1834131 (PMC13259776; doi:10.3389/fnut.2026.1834131)
Supplement: Supplementary file 1 [file Table_1.docx]

**Table S1.** Components and Scoring Criteria of the OBS

| **Category** | **Variable** | **Scoring Criteria (0–2)** |
| --- | --- | --- |
| **Antioxidant Factors (n=14)** |  |  |
| Nutrients (based on energy-adjusted intake, sex-specific tertiles) | Vitamin C, Vitamin E, Selenium, β-Carotene, α-Carotene, Lutein + Zeaxanthin, Lycopene, Vitamin A, Folate, PUFA (polyunsaturated fatty acids), Fiber | Tertile 1 = 0  Tertile 2 = 1  Tertile 3 = 2 |
| Lifestyle | Physical activity | Low = 0  Moderate = 1  High = 2 |
| Diet | Iron intake | Tertile 1 = 2  Tertile 2 = 1  Tertile 3 = 0 |
| Diet | Caffeine intake | Tertile 1 = 0  Tertile 2 = 1  Tertile 3 = 2 |
| **Pro-oxidant Factors (n=6)** |  |  |
| Lifestyle | Smoking status | Current = 0  Former = 1  Never = 2 |
| Lifestyle | Alcohol consumption | Heavy = 0  Moderate = 1  None/light = 2 |
| Nutrients | Saturated fat intake | Tertile 1 = 2  Tertile 2 = 1  Tertile 3 = 0 |
| Nutrients | Total fat intake | Tertile 1 = 2  Tertile 2 = 1  Tertile 3 = 0 |
| Nutrients | Cholesterol intake | Tertile 1 = 2  Tertile 2 = 1  Tertile 3 = 0 |
| Biomarker | Serum ferritin | Tertile 1 = 2  Tertile 2 = 1  Tertile 3 = 0 |
